# Diagnostic tools in the differential diagnosis of giant cell-rich lesions of bone at biopsy

**DOI:** 10.18632/oncotarget.25725

**Published:** 2018-07-10

**Authors:** Jan Rehkämper, Konrad Steinestel, Birte Jeiler, Sandra Elges, Elena Hekeler, Sebastian Huss, Jan Sperveslage, Jendrik Hardes, Arne Streitbürger, Georg Gosheger, Eva Wardelmann, Daniel Baumhoer, Marcel Trautmann, Wolfgang Hartmann

**Affiliations:** ^1^ Gerhard-Domagk-Institute of Pathology, University Hospital Münster, Münster, Germany; ^2^ Institute of Pathology and Molecular Pathology, Bundeswehrkrankenhaus Ulm, Ulm, Germany; ^3^ Department of Orthopaedics and Tumor Orthopaedics, University Hospital Münster, Münster, Germany; ^4^ Bone Tumor Reference Centre, Institute of Pathology, University Hospital Basel, University of Basel, Basel, Switzerland

**Keywords:** giant cell tumor of bone, chondroblastoma, aneurysmal bone cyst, H3F3A/B, diagnostic algorithm

## Abstract

The diagnosis of giant cell-rich lesions of bone can be challenging if radiological findings are ambiguous and tissue of the biologically deciding component is underrepresented in biopsy specimens. The frequent association of giant cell tumor of bone (GCT) and chondroblastoma (CB) with (secondary) aneurysmal bone cysts (ABC) may further impede correct classification. The present study evaluates the potentials and limitations of mutation-specific histone H3.3 and DOG1 immunohistochemistry, Sanger-/next generation sequencing (NGS) and FISH analysis in the differential diagnosis of 23 GCT, 14 CB and 19 ABC. All morphologically typical GCT and CB harbored mutations in the *H3F3A* or *H3F3B* gene, respectively. These were, except for one uncommon G34L mutation in a GCT, reliably and specifically detected by mutation-specific H3.3 G34W or H3.3 K36M immunohistochemistry and DNA sequencing. In the diagnostic substantiation of CB, DOG1 staining was less sensitive compared to H3.3 K36M immunohistochemistry. 47% of ABC specifically showed translocations of the *USP6* gene, while mutations in *H3F3A/B* were absent.

Based on the results of this study, we conclude that mutation-specific H3.3 immunohistochemistry (selectively complemented with NGS-based DNA sequencing) and *USP6* FISH analysis enable a reliable diagnostic distinction of GCT, CB and ABC of morphologically and radiologically difficult cases.

## INTRODUCTION

Diagnosis of giant cell-rich lesions of bone is usually based on a combination of morphological and radiological features. This interdisciplinary approach has proven particularly helpful with regard to lesions that may display a significant overlap of radiographic and morphological features, as is the case for aneurysmal bone cysts (ABC), giant cell tumor of bone (GCT) and chondroblastoma (CB). However, cases in which specific primary bone tumors are disguised by a (dominant) ABC component may still elude correct diagnostic classification, particularly if tissue of the underlying biologically leading tumor is underrepresented in the biopsy specimen.

ABC is a benign expansive lytic lesion of bone composed of multiple blood-filled cysts that are separated by fibrous septa containing variable numbers of lesional fibroblast-like cells as well as osteoclast-like giant cells. Traditionally, ABC is classified into primary ABC and secondary ABC [1]. Primary ABC has recently been proven to represent an original neoplastic process [2], while secondary ABC develops in association with an underlying benign or malignant bone tumor, including GCT and CB [3–5].

GCT of bone is most frequently located in the epi-metaphyseal region of long bones [6, 7]. In most cases, GCT is a benign lesion with a locally aggressive behavior, however, rarely metastases to the lungs may occur [8, 9]. Histologically, GCTs is characterized by a cytologically bland spindle cell population intermingled with osteoclast-like giant cells and macrophages. In GCT, the monoclonal antibody Denosumab represents a specific therapeutic option which is based on the inhibition of intralesional receptor activator of nuclear factor κ-B (RANK)-signals driving the recruitment of the osteoclast-like giant cells [10, 11], thus preventing further bone destruction and inducing lesional consolidation [12, 13].

CB is typically related to the growth plate of long bones with involvement of the epiphysis or apophysis [3, 14]. Histologically, CB is characterized by an immature chondroid matrix and uniform polygonal cells with round to ovoid nuclei interspersed with osteoclast-like giant cells [1].

Though the specific clinical, radiological and histological features of these three lesions are most often evident, the exact histological classification, even in open biopsy specimens, may be difficult due to a significant morphological overlap. From the clinical perspective, exact diagnostic typing is elementary: As ABC, GCT and CB of bone most frequently represent locally aggressive lesions, the therapeutic concept for all of them is mainly based on local removal e.g. by curettage. However, in contrast to ABC, GCT (and even CB) may rarely spread, particularly to the lung, making an adequate staging of the patient essential. Beyond that, GCT may therapeutically benefit from treatment with Denosumab, which is not an established treatment for CB or ABC. Eventually, rare cases of malignant GCT of bone have been described.

While a subset of primary ABC is molecularly characterized by *USP6* translocations [15], GCT and CB have recently been shown to carry *H3F3A* (G34W) or *H3F3B* (K36M) gene mutations, respectively [16]. Mutational analysis of *H3F3A* and *H3F3B* has therefore been successfully introduced as specific tool in routine diagnostics [17]. Beyond that, two monoclonal mutation-specific antibodies detecting the most common mutations in histone variants H3.3 have recently been developed [18, 19]. In morphologically difficult cases, specific immunohistochemical and molecular analyses may therefore contribute substantially to the final diagnostic characterization. Diagnostic problems may still arise, however, if tissue of the biologically leading tumor is underrepresented in the biopsy specimen and if a (secondary) ABC component represents the dominant morphological feature.

The present study was performed to comparatively evaluate the potential of different diagnostic approaches. To this end, *H3F3A/B* mutational testing using next generation sequencing (NGS) and Sanger DNA sequencing were comparatively performed with immunohistochemical stainings applying mutation-specific histone H3.3 antibodies, complemented with immunostainings against DOG1 as a marker of CB [20] and *USP6-*break apart fluorescent *in situ* hybridization (FISH) for the characterization of ABC. Based on these analyses, we were able to substantiate diagnoses even in morphologically challenging giant cell-rich lesions of bone.

## RESULTS

**Figure 1:**
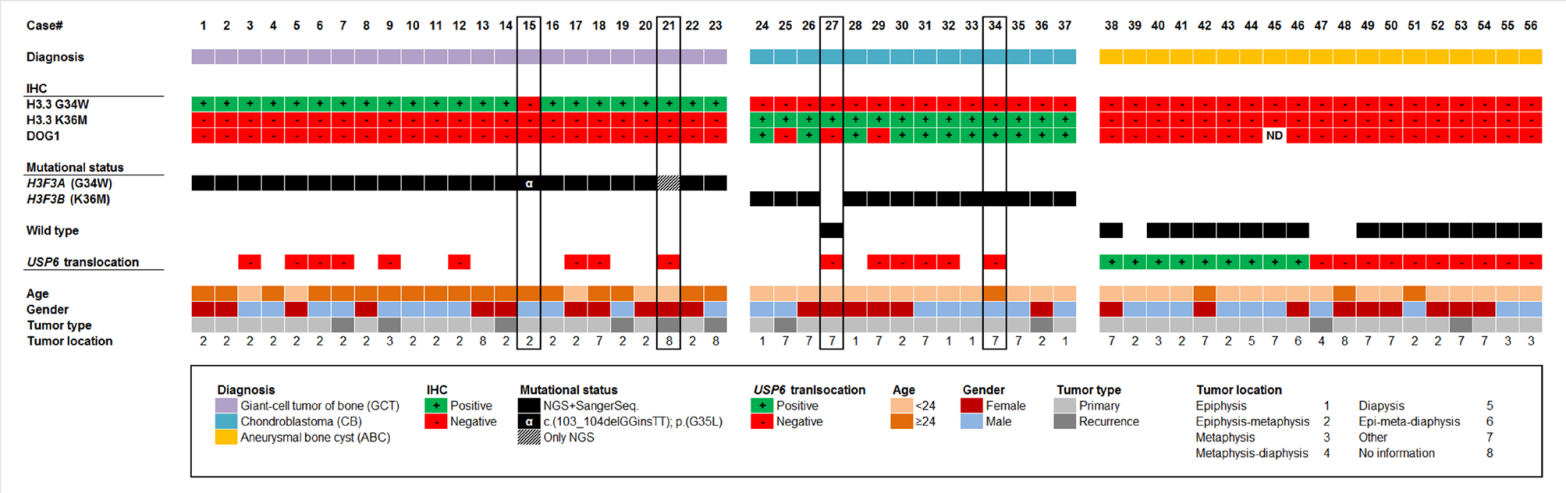
Clustered overview of clinical and pathological patient data, the results of the immunostainings (H3.3 G34W, H3.3 K36M, DOG1), the mutational *H3F3A/B* status as detected by DNA sequencing and the result of the USP6 break-apart FISH Cases are categorized in diagnosis groups (GCT, CB, ABC).

**Figure 2:**
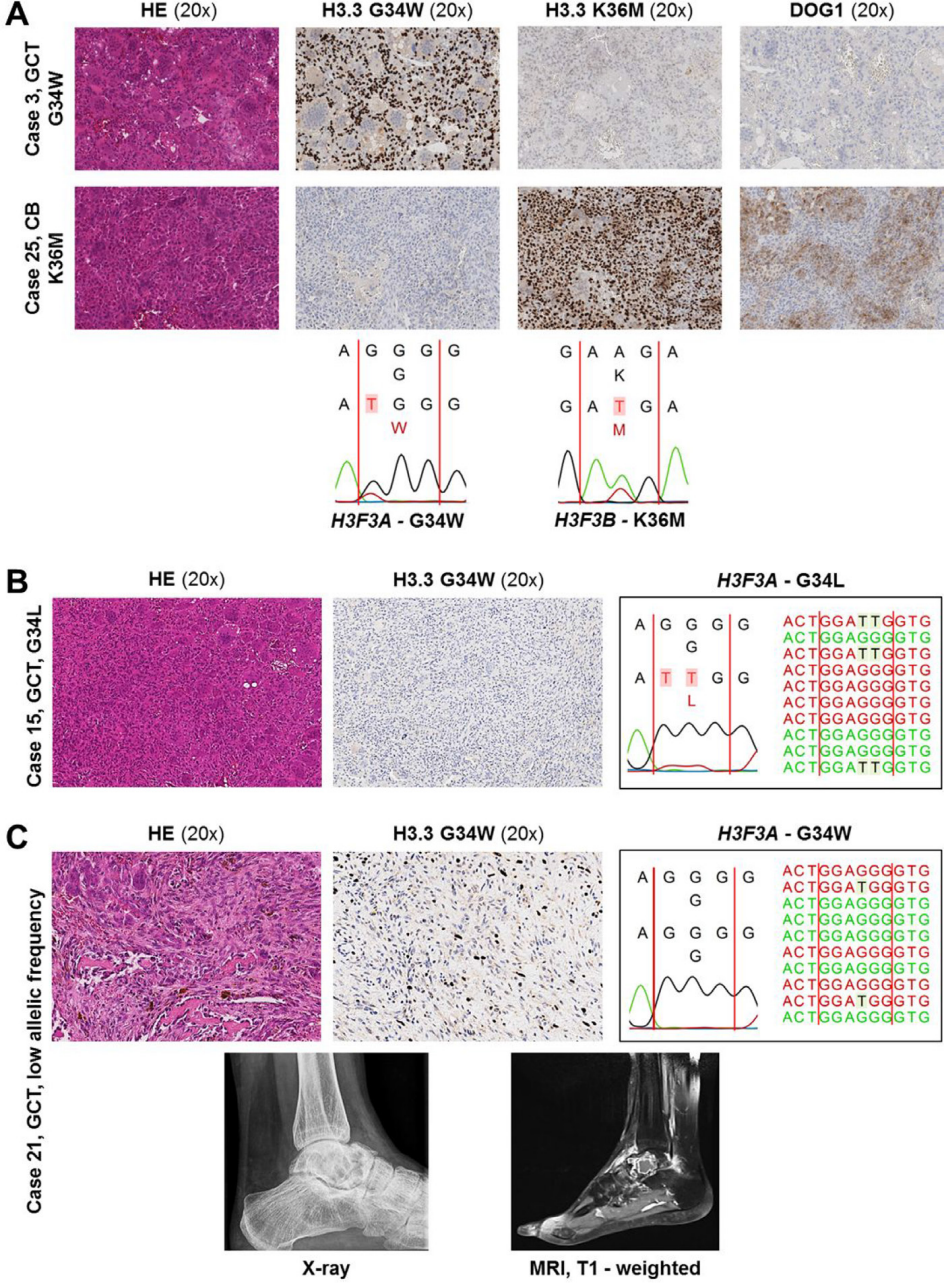
(**A**) Representative staining results and DNA sequence electropherograms. Top: GCT with G34W alteration (case #3). Bottom: CB with K36M alteration (case #25). Corresponding DNA sequence electropherograms are shown below. (**B, C**) Immunohistochemical staining and corresponding Sanger and NGS sequencing results in two diagnostically challenging cases of GCT. (B) GCT with a G34L alteration (case #15). (C) Top: GCT with low mutation frequency (7.5%) demonstrated by immunohistochemistry and NGS, not detectable by Sanger sequencing. Bottom: X-ray and MRI image (case #21).

All morphologically typical GCT and CB showed mutations in the *H3F3A* (G34W or G34L) and *H3F3B* (K36M) genes, respectively. The type of mutation detected by DNA sequencing correlated with the immunohistochemical staining using the mutation-specific antibodies against H3.3 G34W and H3.3 K36M (Figures 1 and 2A). In one case (#21) detection of the G34W alteration by sequencing was restricted to NGS due to the low content of lesional cells despite macrodissection (7.5% mutation frequency) (Figures 1 and 2B). In one case of GCT (#15), immunohistochemical staining with the G34W antibody was negative, however, NGS and Sanger DNA sequencing revealed an uncommon G34L mutation in this case (Figures 1 and 2B).

**Figure 3:**
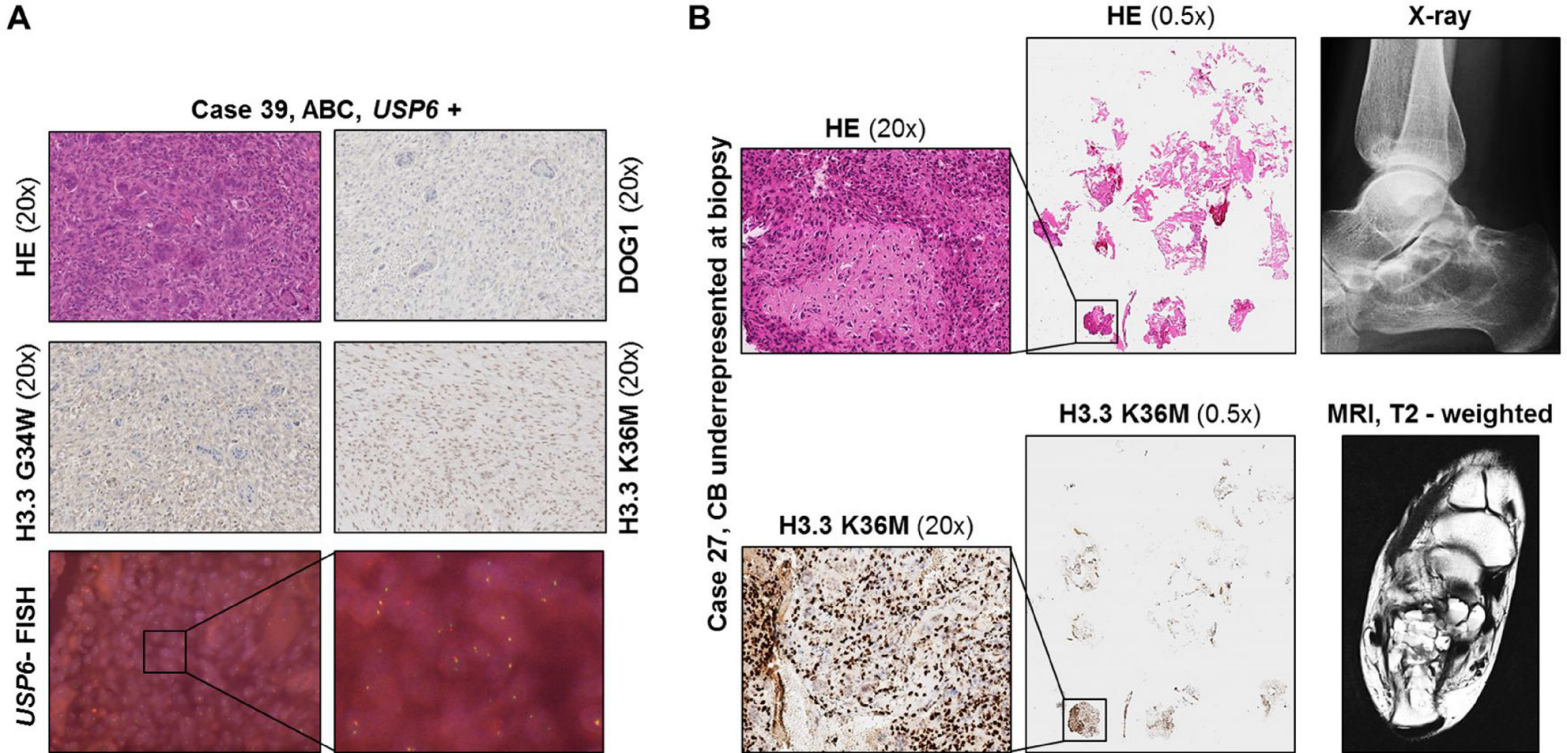
(**A**) Immunohistochemical staining and *USP6* FISH results shown for primary ABC (case #39). (**B**) Left: Immunohistochemical H3.3 K36M staining results in a case with extensive secondary ABC and underrepresented lesional CB tissue in the biopsy material (case #27). No *H3F3B* mutation could be detected by DNA sequencing due to the low fraction of mutated lesional cells. Right side: X-ray and MRI image.

A subset of 9/19 radiographically and morphologically typical ABC showed translocations of the *USP6* gene locus. None of the ABC harbored mutations in the *H3F3A/B* genes, either by Sanger or NGS sequencing. Immunohistochemistry with mutation-specific antibodies revealed no positive staining reactions (Figures 1 and 3A). DOG1 staining was negative in all cases of ABC (Figure 1).

Two cases (#27, #34) radiographically interpreted as ABC and histologically showing a predominant membrane-like fibrohistiocytic pattern focally stained positive with the mutation-specific H3.3 K36M antibody (Figure 3B). In case #27, no mutation could be detected using both NGS and Sanger sequencing due to the low fraction of mutated lesional cells.

DOG1 staining was evaluated as positive in 11/14 cases of CB (100% specificity, 78.5% sensitivity). There was no immunoreactivity with the antibody against DOG1 in any of the cases of GCT and ABC, respectively (Figure 1).

The clinicopathological characteristics as well as the mutational and immunohistochemical profiles of all analyzed 56 cases are summarized in Figure 1 and Table 1.

**Table 1:** Clinicopathological characteristics of all cases included in the study cohort

No. of cases	*N*
Total	56
GCT	23 (41.1%)
CB	14 (25%)
ABC	19 (33.9%)
**Age** (years)	**mean (±SD), median, range**
Total	23.8 (±12.2), 20, 5–72
GCT	30.6 (±8.6), 30, 14–45
CB	18.4 (±4.1), 19.5, 12–26
ABC	19.4 (±15.5), 16, 5–72
**Sex**	**Male/Female (%)**
Total	30/26 (53.6/46.4)
GCT	12/11 (52.2/47.2)
CB	8/6 (57.1/42.9)
ABC	10/9 (52.6/47.4)
**Tumor type**	**Primary/Recurrence (%)**
Total	46/10 (82.1/17.9)
GCT	17/6 (73.9/26.1)
CB	12/2 (85.7/14.3)
ABC	17/2 (89.5/10.5)

Abbreviation: *GCT*, Giant cell tumor of bone; *CB*, Chondroblastoma; *ABC*, Aneurysmal bone cyst.

## DISCUSSION

GCT and CB are giant cell-rich lesions of bone which are frequently associated with the formation of secondary ABC. While these lesions are most often well distinguishable through typical radiological and histological appearance in a given clinical setting, differential diagnosis can sometimes be challenging. This is due to a certain morphological overlap which is of minor relevance in ample material of curettage where histological patterns can easily be discerned. However, it can become an issue in biopsies which may contain only few lesional cells and in which interpretation may be further impeded through predominant sampling of an obscuring (secondary) ABC.

The DNA synthesis-independent replacement histone H3.3 is encoded by two different genes, *H3F3A* and *H3F3B.* Tumor-type specific mutations have been demonstrated in pediatric glioma, CB and GCT, qualifying them as driver mutations. While in pediatric glioma and glioblastoma K27M and G34R/V variants occur in *H3F3A*, GCT harbor mainly *H3F3A* G34W alterations whereas CB display predominantly K36M mutations in *H3F3B* [16, 21]. While the functional impact of these alterations in CB and GCT is not understood in detail, data from childhood brain tumors imply that the K27M mutation in *H3F3A* mutation acts via a dominant negative gain of function by inhibition of the methyltransferase activity of EZH2, thus abolishing Polycomb-mediated repression of numerous genes [22]. Concerning the functional significance of the G34R/V in childhood brain tumors it has been hypothesized that the proximity of H3G34 and H3K36 might play a major role: H3K36 represents a residue that regulates transcriptional elongation, and H3G34R/V mutant nucleosomes have been shown to carry reduced methylation of H3K36 by SETD2, the only human methyltransferase specific for H3K36 [22]. This suggests that the H3G34R/V mutation impacts the ability of histone-modifying complexes to methylate H3K36, thus altering the transcription of several target genes. It is unclear in how far these insights are transferable to the bone lesions analyzed here, but similar pathogenic mechanisms appear probable.

The reported frequency of *H3F3A* mutations (G34W) in GCT ranges from 85–96% [23, 24], making the assessment of *H3F3A* mutational status a reliable diagnostic tool. A recently developed monoclonal antibody targeting the typical H3.3 G34W mutation was described as a sensitive and specific marker for diagnosis [19, 25]. In our study, all 23 GCT harbored mutations in *H3F3A*. While the majority of the mutations was detectable employing Sanger sequencing, NGS sequencing (in full accord with the immunohistochemical result) revealed one additional mutation-positive case (#21) with low mutation frequency, underlining the diagnostic value of NGS in samples with low content of lesional cells. The immunohistochemical approach led to a correct diagnosis in 36/37 cases of GCT and CB. In the single case (#15) of a H3.3 G34W IHC-negative GCT specimen, a rare G34L mutation could be detected by both DNA sequencing techniques (Figure 2B). This is in line with the findings reported by Amary *et al.* (2017) who screened 235 GCT and found nuclear immunostaining with the H3.3 G34W antibody in 213 cases. In 6 cases, IHC staining was negative due to underlying G34L, G34M or G34V *H3F3A* alterations. Therefore, since the immunohistochemical approach in GCT is limited to the G34W mutation, a small number of cases of GCT carry the risk to be misdiagnosed based on immunohistochemistry alone. By performing (additional) DNA sequencing in case of suggestive morphology and negative H3.3 G34W immunohistochemistry, uncommon H3.3 alterations can also be detected, with NGS being the advantageous technique due to its higher sensitivity. It has to be emphasized that the differential diagnosis of a malignant tumor associated with a secondary ABC should always be considered in these patients, particularly since rare H3.3 hotspot mutations in malignant bone tumors, especially osteosarcomas, have been described previously [25–27], and since few cases of osteosarcoma have been reported with immunoreactivity for the G34W mutation-specific antibody; however, immunoreactive tumor cells displayed clear cytological atypia in these cases [25].

CB is described to harbor *H3F3* mutations resulting in K36M alterations in 95% of cases. In most cases, mutations are found in *H3F3B* with only very few alterations detected in *H3F3A* *[16]*. The monoclonal H3.3 K36M antibody has been shown to be highly specific for the diagnosis of CB [18]. In the present study 13/14 CB harboured the typical *H3F3B* K36M mutation detected by both DNA sequencing methods. The immunohistochemical approach led to a correct diagnosis in 14/14 cases using the H3.3 K36M antibody and in 11/14 cases using DOG1 immunostaining. Discovered on gastrointestinal stromal tumours 1 (DOG1) was initially described as a highly expressed calcium-dependent chloride channel in gastrointestinal stroma tumors (GIST) [28] and shown to serve as a sensitive diagnostic marker, particulary in *PDGFRA*-mutated tumors (wild-type for *KIT*) [29]. In a study assessing different immunohistochemical markers in CB, Akpalo and colleagues then identified DOG1 to specifically stain nests of chondroblasts in CB, qualifying as a diagnostic marker [20]. While in GIST, DOG1 appears to play a particular functional role by downregulation of insulin-like growth factor-binding protein 5 (IGFBP5), a potent antiangiogenic factor [30], its functional role in CB is not understood. In the present study, we compared the diagnostic potential of DOG1 immunohistochemistry and the mutation-specific H3.3 K36M antibody.

In one case (#27), it was not possible to detect the mutation by DNA sequencing due to underrepresented mutated lesional cells in the biopsy material. However, immunohistochemistry revealed a small area with distinct nuclear staining indicating a K36M mutation in at least few cells in that case (Figure 3B). DOG1 immunostaining was negative in this case. Interestingly, in this study, a small number of cases that had radiographically been interpreted as ABC and that, in the biopsy material, showed a predominantly membrane-like fibrohistiocytic pattern fitting with ABC, turned out to be (underrepresented) CB with (predominant) secondary ABC as indicated by the molecular phenotype (Figure 3B, case #27).

More than 65% of primary ABC have been shown to carry clonal rearrangements of the *USP6* gene locus, indicating a true neoplastic nature of these lesions [31]. In the present study, 9/19 (47.3%) ABCs displayed *USP6* chromosomal translocations, qualifying these cases as primary ABC. The somewhat minor frequency of *USP6* translocations in our group of (primary) ABC compared to what was reported by Oliveira and colleagues [31] is probably due to the relatively low ABC sample numbers included in our study. Since among the ABC cases without rearrangement of the *USP6* gene morphologically undetected components of GCT and CB might be present, *H3F3A/B* mutational status was assessed. DNA sequencing and immunohistochemistry (H3.3 mutation-specific antibodies, DOG1) of all 19 ABC cases included in our study revealed no mutation in *H3F3A/B* and showed no positive IHC staining, ruling out secondary ABC on the basis of GCT or CB.

In the present study we correlated the results from mutation-specific immunohistochemical tissue typing with results from two different DNA sequencing techniques in giant cell-rich lesions of bone. We provide evidence that ambiguous cases with no clear-cut radiological and morphological appearance can reliably be diagnosed by the use of H3.3 mutation-specific immunohistochemistry and *USP6* FISH as diagnostic tools. If diagnosis is not possible with certainty, DNA sequencing should be performed to detect uncommon *H3F3A/B* mutations that have been described especially in GCT. Considering the sequencing approach, NGS represents the more sensitive technique to be employed, especially if a low mutational frequency needs to be detected.

**Figure 4:**
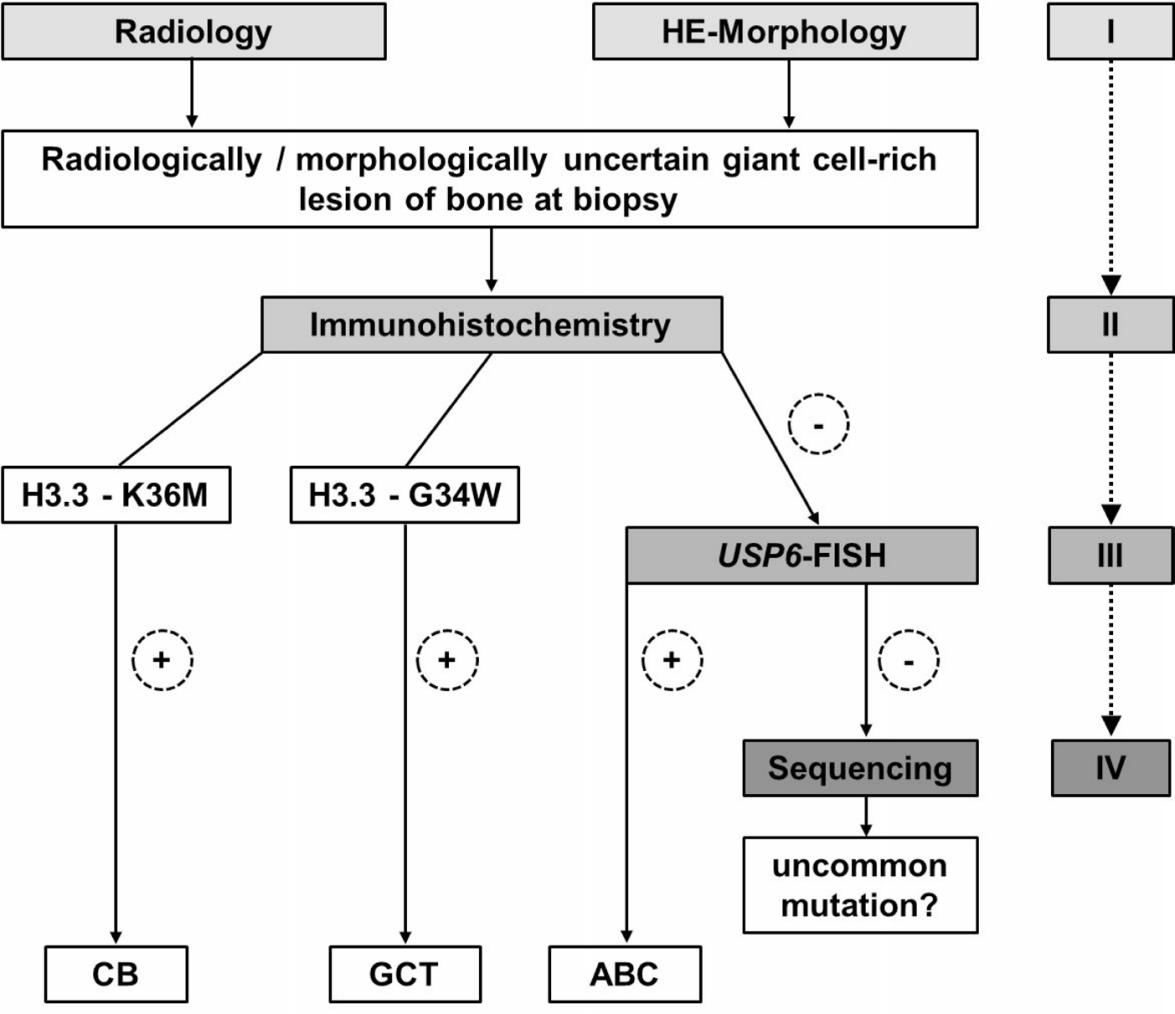
Proposed diagnostic algorithm for radiologically and morphologically ambiguous giant cell-rich lesions of bone at biopsy: Immunohistochemistry with mutation-specific H3.3 antibodies is recommended as a starting point for the detection of underlying GCT or CB In case of negativity, *USP6* FISH is suggested to confirm/exclude primary ABC. DNA sequencing of the *H3F3A* and *H3F3B* genes is required only in immunohistochemically H3.3 G34W- and K36M-negative and non *USP6*-rearranged cases to rule out secondary ABC with underlying GCT or CB with a non-typical *H3F3A* and/or *H3F3B* gene alteration.

We propose a diagnostic algorithm for small biopsies of diagnostically ambiguous giant cell-rich lesions of bone starting with morphological evaluation in correlation with radiological findings (step I), followed by immunohistochemistry with mutation-specific H3.3 (G34W, K36M) antibodies (step II) and *USP6* FISH analysis (step III) in cases negative with both mutation-specific antibodies. DNA sequencing (employing NGS as the more sensitive technique) of the *H3F3A* and *H3F3B* genes (step IV) is only necessary in cases negative in H3.3 G34W or K36M immunohistochemistry and lacking a *USP6* rearrangement, which might point either to a GCT or CB with uncommon mutations or a *USP6*-negative ABC (Figure 4). This diagnostic algorithm should help to avoid underdiagnoses of CB and GCT especially in cases with underrepresented tissue of the biologically leading component in biopsy material. This is of importance with regard to the required clinical staging and Denosumab as a therapeutic option in GCT.

## MATERIALS AND METHODS

### Cohort

In total, 23 GCT, 14 CB and 19 ABC analyzed in the routine diagnostics of the Gerhard-Domagk-Institute of Pathology and of the Basel Bone Tumor Reference Centre, Switzerland, were included in the study. Diagnoses were based on the synopsis of radiographic features, morphological and molecular findings. In the GCT cohort, mean age was 30.6 (±8.6) years (median, 30.0 years). A subset of 6/23 GCT was classified as recurrence following clinical data. Mean age in the CB group was 18.4 (±4.1) years (median, 19.5 years). Two of the lesions presented as local recurrences. Within the ABC group, two cases were categorized as local recurrences. Mean age was 19.4 (±15.5) years (median, 16.0 years). Approval of the study was obtained from the local Ethical Committees in Münster and Basel (reference no. 2017-310-f-S and reference 274/12, respectively).

### DNA sequencing

The mutational status was analyzed by Sanger- and next generation amplicon based DNA sequencing (NGS). A customized GeneRead DNAseq Mix-n-Match V2 panel (Qiagen, Hilden, Germany) was applied to simultaneously amplify the mutational hot spot regions of 19 cancer-related genes. Target enrichment was processed by means of the GeneRead DNAseq Panel PCR V2 Kit (Qiagen, Hilden, Germany), following the manufacturer’s instructions. All purification and size selection steps were performed employing Agencourt AMPure XP magnetic beads (Beckman Coulter, Brea, CA, USA). End repair, A-addition and ligation to NEXTflex-96 DNA barcodes (Bioo Scientific, Austin, Texas, USA) were conducted using the GeneRead DNA Library I Core Kit (Qiagen, Hilden, Germany). Amplification of adapter-ligated DNA was performed using NEXTflex primers (Bioo Scientific, Austin, TX, USA) and the HiFi PCR Master Mix (GeneRead DNA I Amp Kit, Qiagen). NGS was performed applying 12.5 pM library pools (2% PhiX V3 control) and the MiSeq Reagent v2 chemistry (Illumina, San Diego, CA, USA). NGS data analysis was performed by means of the CLC Biomedical Genomics Workbench software (CLC bio, Qiagen, Hilden, Germany). Additional validation of the *H3F3A/B* mutational status was performed by Sanger sequencing according to standard procedures using the BigDye Terminator v3.1 Cycle Sequencing Kit (Life Technologies, Carlsbad, CA, USA). Following primer sets were applied: (I) *H3F3A:* 5′GTC TCT GTA CCA TGG CTC GT-3′ (for) and 5′ACA AGA GAG ACT TTG TCC CAT TT-3′ (rev) and (II) *H3F3B*: 5′TCT TCG GGG CGT CTT TCT TA-3′ (for) and 5′GAG CAG GGG AGG AGT GAG-3′ (rev).

### Immunohistochemistry (IHC)

IHC was performed on 3µm-thick sections from paraffin-embedded tissue using an automated immunohistochemistry staining system (Ventana BenchMark ULTRA, Ventana Medical Systems, Tucson, AZ, USA). Two monoclonal rabbit antibodies detecting the mutated H3.3 histones (G34W, Clone RM263 and K36M, Clone RM192) were purchased from RevMab (RevMab Biosciences, San Francisco, CA, USA). Both staining protocols were established using formalin-fixed and paraffin-embedded tissue slides from GCT and CB cases with known mutational status. A monoclonal DOG1 antibody was purchased from Cell Marque (clone SP31, Cell marque, Rocklin, CA, USA). In brief, sections were deparaffinized and pre-treated with Cell Conditioning 1 or 2 (CC1/CC2, Ventana, AZ, USA) for 32 minutes at 95° C (H3.3 G34W, H3.3 K36M) or 64 minutes at 100° C (DOG1). The primary antibodies G34W and K36M H3.3 were then incubated for 32 minutes at 36° C (dilution 1:500) and DOG1 for 16 minutes at 36° C. Immunoreactions were visualized using the Ventana Optiview DAB IHC detection kit (REF 760–700, Ventana, AZ, USA). The sections were finally counterstained with haematoxylin (REF 790–2208, Ventana, AZ, USA) and bluing reagent (REF 760–2037, Ventana, AZ, USA). Nuclear stainings with the H3.3 monoclonal antibodies were evaluated as *positive* or *negative*. DOG1 was graded as absent, weak, moderate or strong cytoplasmatic/membranous staining (0–3) as published by Akpalo *et al.* [20]. Weak (0) and moderate (1) staining results were finally evaluated as *negative* while moderate and strong stainings were evaluated as *positive.*

### Fluorescence *in situ* hybridization (FISH)

The *USP6* dual-colour break-apart probe (Zytovision, Bremerhaven, Germany) was used for analysis of the *USP6* gene locus, employing the criteria published by Oliveira and colleagues [15]. FISH protocol was performed as described before [32].
